# Longitudinal Changes of Peripheral Blood DC Subsets and Regulatory T Cells in Chinese Chronic HIV-1-Infected Patients during Antiretroviral Therapy

**DOI:** 10.1371/journal.pone.0037966

**Published:** 2012-05-31

**Authors:** Mei Zhang, Hongwei Zhang, Tong Zhang, Yunxia Ji, Yanmei Jiao, Hao Wu

**Affiliations:** Center for Infectious Diseases, Beijing You'an Hospital, Capital Medical University, Beijing, China; University of Ottawa, Canada

## Abstract

It has been emphasized that chronic generalized immune dysfunction is the leading event in the pathogenesis of HIV infection, in which the contribution of dendritic cells (DCs) and regulatory T cells (Tregs) should not be underestimated. In current study, we assessed the longitudinal changes of peripheral blood DC subsets and Tregs in chronically asymptomatic treatment-naive HIV-1-infected patients during 60 weeks of antiretroviral therapy (ART), and compared with those in healthy controls and long term non-progressors (LTNPs). Blood samples were collected at week 0, 4, 12, 24, 48 and 60 of treatment to measure the counts of DC subsets and Tregs by flow cytometry and IFN-a plasma levels by ELISA. The counts of myeloid dendritic cells (mDCs) increased during ART, reaching similar levels to healthy controls at week 60 post ART but still lower than those of LTNPs. In HIV-1-infected patients, the mDCs counts were directly correlated with CD4 counts during ART. Changes in mDCs at week 8 were positively correlated with the changes in CD4 counts at week 60 post ART. However, the counts and function of plasmacytoid dendritic cells (pDCs) remained relatively stable during ART, and similar to those in healthy controls and LTNPs. The percentage of Tregs increased before ART and normalized after ART. Importantly, we found pDCs counts were associated with percentage of Tregs during ART, which may help in understanding of the role of these cells in HIV infection.

## Introduction

HIV infection is characterized with an initial, occasionally symptomatic acute phase followed by an asymptomatic period of variable length culminating in clinically evident immunodeficiency [1]. Recent findings have emphasized that chronic generalized immune dysfunction is the leading event in the pathogenesis of HIV infection, in which the contribution of dendritic cells (DCs) and regulatory T cells (Tregs) should not be underestimated. DCs are professional antigen-presenting cells required for generation of adaptive immunity [2], and Tregs are essential for immune nullipotency and immune suppression via cell-cell contacts or cytokine secretion [3]. There are accumulating evidences that DCs and Tregs may be valuable tools for modulating immunity in the setting of chronic viral infections, and interactions between them may play a crucial role in the balance of immunity and tolerance.

DCs are at the interface of innate and adaptive immunity, which specialize in the initiation of adaptive immune responses to invading pathogens such as HIV [4]. In human peripheral blood, two main subsets have been identified according to their different phenotypes and functions: myeloid and plasmacytoid DCs [5]. Myeloid DCs (mDCs) are more frequently found, which sense both bacterial and viral pattern motifs through a broader range of TLRs and secret high levels of IL-12 in response to activation [2]. Plasmacytoid DCs (pDCs), representing 0.2–0.8% of peripheral blood mononuclear cells (PBMCs), selectively express Toll-like receptor (TLR)-7 and TLR9, and are the most potent IFN-a-producing cells in the body following viral stimulation [6]. The role of DCs in HIV infection is still under debate because HIV has evolved subtle strategies to hijack key cellular components in DCs, leading to viral acquisition and dissemination while dampening or delaying antiviral responses.

Tregs, representing approximately 5–10% of CD4+T cells in the peripheral blood, profoundly inhibit T-cell activation, proliferation and effector function, and play a role in the regulation of chronic viral infections, including HIV. Several types of Tregs have been characterized, most prominently natural and inducible CD4+CD25+Tregs [7]. So far, Tregs are identified by expression of a number of molecules, including the alpha chain of the IL-2 receptor (CD25), cytotoxic T-lymphocyte associated protein 4 (CTLA-4), glucocorticoid-induced tumor necrosis factor receptor (GITR) and transcription factor forkhead box P3 (Foxp3) [7], [8], among which FoxP3 is proposed as an accurate marker for Tregs. The role of Tregs in HIV infection is also paradoxical. On the one hand, Tregs downregulate immune responses and limit the magnitude of effector responses, resulting in failure to control HIV infection. On the other hand, they may also suppress chronic immune activation, and thus protect from HIV progression [8].

Significant depletion and functional impairment of DCs have been documented in HIV infection [9]–[12], but the mechanisms underlying remain to be elucidated. Since the advent of highly active antiretroviral therapy (HAART), HIV-infected patients have experienced a significant delay in disease progression and longer life expectancy. However, the impact of ART on DCs loss or recovery in HIV infection is unclear, and few studies have evaluated the longitudinal changes of peripheral blood DC subsets. Several studies indicate that ART is not effective at increasing blood mDCs [10], [13], [14], while others suggest that ART significantly restores blood mDCs numbers [9], [12], [15]. As for the changes of pDCs number and function after ART, there has been similar controversy [9], [10], [12], [14], [15].

Nowadays, numerous studies have demonstrated the involvement of Tregs in HIV infections, whereas, they can be good or evil [8]. Results regarding the influence of ART on percentage and counts of Tregs are not consistent among studies [16]–[21], and longitudinal effects of ART on Tregs are rarely reported.

In this study, we described the longitudinal changes of peripheral blood DC subsets and Tregs in a group of chronically asymptomatic treatment-naive HIV-1-infected patients with initial CD4 counts less than 350 cells/ul during 60 weeks of ART, and compared with those in 15 HIV-uninfected healthy controls and 15 long term non-progressors (LTNPs).

## Results

### Viral loads and CD4+T cells counts before and after initiation of ART

HIV-1 RNA viral loads (VLs) and CD4 counts were measured in all 17 HIV-1-infected patients at week 0, 4, 8, 12, 24, 48 and 60 of ART (Table 1). VLs and CD4 counts were also measured in LTNPs and CD4 counts in healthy controls for comparison. In HIV-1-infected patients, VLs decreased significantly since initiation of ART. Similarly, CD4 counts were markedly increased. In healthy controls and LTNPs median CD4 counts (748 cells/ul and 693 cells/ul) were significantly higher than those in HIV-1-infected patients at both week 0 (*p*<0.001) and week 60 (*p*<0.001).

**Table 1:** Clinical characteristics of the 17 HIV-1-infected patients.

	Age		VL(copies/ml)							CD4+T cell counts(cells/ul)						
NO.	(years)	ART	w0	w4	w12	w24	w60	Sex	Tr	w0	w4	w8	w12	w24	w48	w60
**1**	42	AZT+3TC+NVP	6158	41	44	<40	<40	M	Ho	25	117	190	187	174	190	265
**2**	29	D4T+3TC+NVP	24185	533	62	118	44	M	Ho	244	210	187	231	330	308	257
**3**	32	D4T+3TC+NVP	5089	352	<40	73	<40	F	ND	248	302	417	385	394	412	532
**4**	31	D4T+3TC+NVP	68639	43701	ND	133	<40	M	Ho	196	416	294	302	322	407	380
**5**	33	AZT+3TC+NVP	48808	1490	341	<40	<40	M	Ho	159	204	251	233	205	418	290
**6**	41	D4T+3TC+NVP	397739	1034	735	59	47	M	Ho	341	400	320	228	334	524	535
**7**	37	D4T+3TC+NVP	1837	257	<40	<40	<40	M	Ho	249	205	306	336	354	384	368
**8**	36	D4T+3TC+NVP	8016	113	<40	<40	<40	M	Ho	227	366	366	344	411	271	363
**9**	30	D4T+3TC+NVP	20206	130	<40	<40	45	M	Ho	116	232	234	136	250	355	371
**10**	27	D4T+3TC+NVP	41811	836	179	<40	<40	M	Ho	267	319	416	473	373	378	490
**11**	28	AZT+3TC+NVP	10416	288	46	71	<40	M	Ho	237	228	260	331	208	350	425
**12**	38	D4T+3TC+NVP	1528	3503	1435	ND	<40	F	He	215	216	235	201	251	247	234
**13**	29	D4T+3TC+NVP	67897	1126	134	<40	<40	M	Ho	190	224	333	270	350	390	341
**14**	28	D4T+3TC+NVP	46227	295	<40	<40	<40	M	Ho	322	308	280	302	290	414	303
**15**	26	D4T+3TC+NVP	20977	578	104	45	<40	M	Ho	66	169	160	193	258	186	170
**16**	42	D4T+3TC+NVP	58578	1655	150	266	<40	M	Ho	124	203	230	285	269	288	310
**17**	30	D4T+3TC+NVP	46746	130	140	<40	<40	M	Ho	34	29	28	41	100	192	167
**Median**	**31**		**24185**	**533** **	**83** **	**<40** **	**<40** **			**215**	**224** **	**260** **	**270** **	**290** **	**355** **	**341** **

ART, antiretroviral therapy; AZT, zidovudine; 3TC, lamivudine; NVP, nevirapine; d4T, stavudine; F, female; M,male; ND, not determined; Tr: transmission route; Ho: homosexual; He: heterosexual;

** *p*<0.01, compared to week 0.

### Dynamics of DCs counts and IFN-a plasma levels during ART

**Figure 1:**
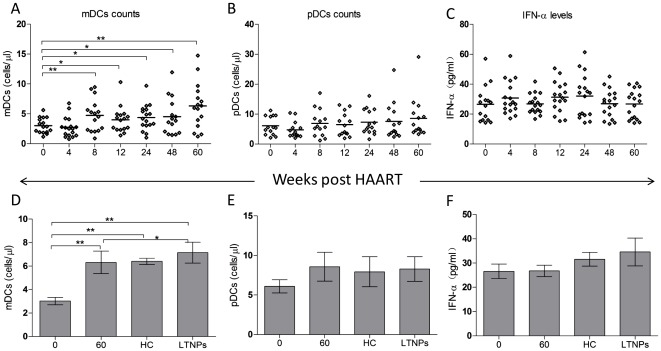
Longitudinal changes in DC subsets and IFN-a plasma levels during ART (weeks). (A–C) Longitudinal changes in counts of mDCs, pDCs and IFN-a plasma levels in HIV-1-infected patients during ART. (D–F) Comparisons of mDCs, pDCs and IFN-a plasma levels among HIV-1-infected patients at week 0, week 60, healthy controls and LTNPs. **p<0.01, *p<0.05.

As shown in Fig. 1, we observed a significantly increase in mDCs after ART, whereas no significant change in pDCs and IFN-a plasma levels (Figure 1). The mDCs counts at w0 in HIV-1-infected patients were significantly lower than those in healthy controls (*p*<0.001) and LTNPs (*p*<0.001). However, the mDCs counts at w60 increased to similar levels in healthy controls (*p* = 0.493) but still lower than those in LTNPs (*p* = 0.014). The counts of pDCs remained relatively stable during ART (6.09cells/ul, 4.75cells/ul, 6.88cells/ul, 6.44cells/ul, 7.29cells/ul, 7.59cells/ul and 8.07cells/ul at week 0, 4, 8, 12, 24, 48, 60 respectively, all *p*>0.05 in comparison to w0) and were not distinguished from those in HIV-1-uninfected controls (7.12cells/ul, both *p*>0.05 as compared to w0 and w60) and the LTNPs (7.23cells/ul, both *p*>0.05 as compared to w0 and w60). To interpret the function of the blood pDCs, we also measured IFN-a plasma levels, and they were not markedly affected by ART (data not shown).

### Association of DCs counts with VLs and CD4 counts

**Figure 2:**
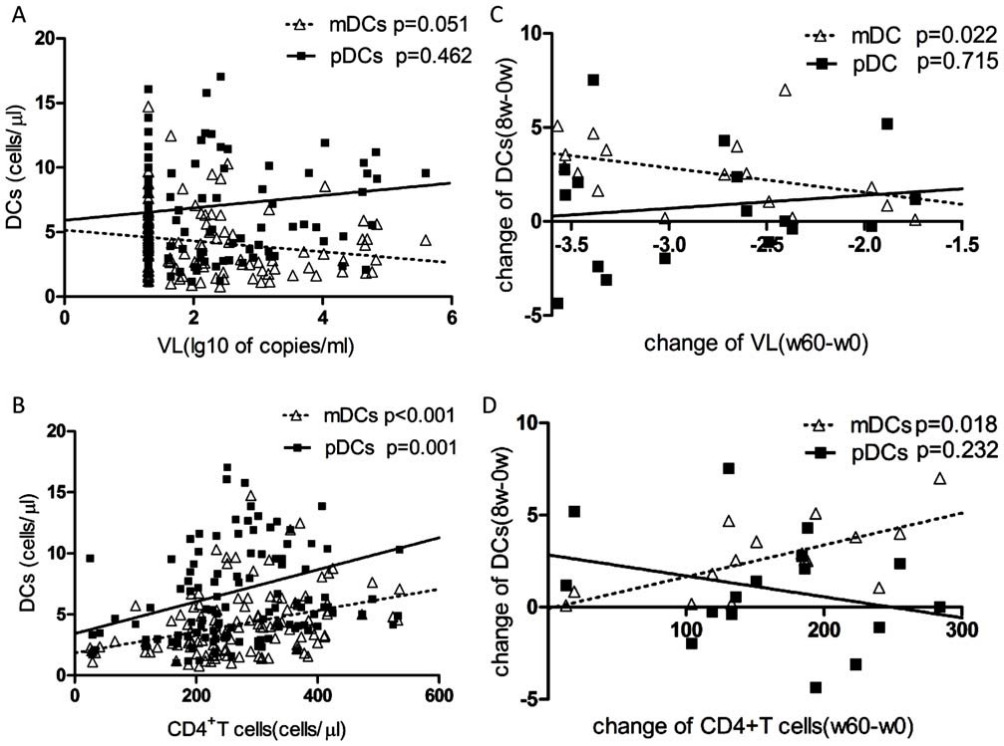
The association of DC subsets with viral loads and CD4+Tcells. (A–B) Correlation between DC subsets counts and plasma viral loads, CD4+Tcells counts during ART. (C–D) Correlation between change in counts of DC subsets at week 8 and the change in plasma viral loads, CD4+Tcells counts at week 60 post ART.

In HIV-1-infected patients, no significant correlations were observed between DC subsets counts and VLs (data not shown). In contrast, a significant positively association were found between mDCs counts and CD4 counts (r = 0.40, *p*<0.001), as well as pDCs counts and CD4 counts (r = 0.319, *p* = 0.001) (Figure 2 A–B). Changes in mDCs at week 8 were negatively correlated with the changes in viral loads at week 60 (r = −0.551, *p* = 0.022) and positively correlated with the changes in CD4 counts at week 60 post ART (r = 0.566, *p* = 0.018) (Figure 2 C–D). However, there were no similar correlations of pDCs with VLs and CD4 counts (data not shown).

### Dynamics of the percentage and counts of Tregs during ART

**Figure 3:**
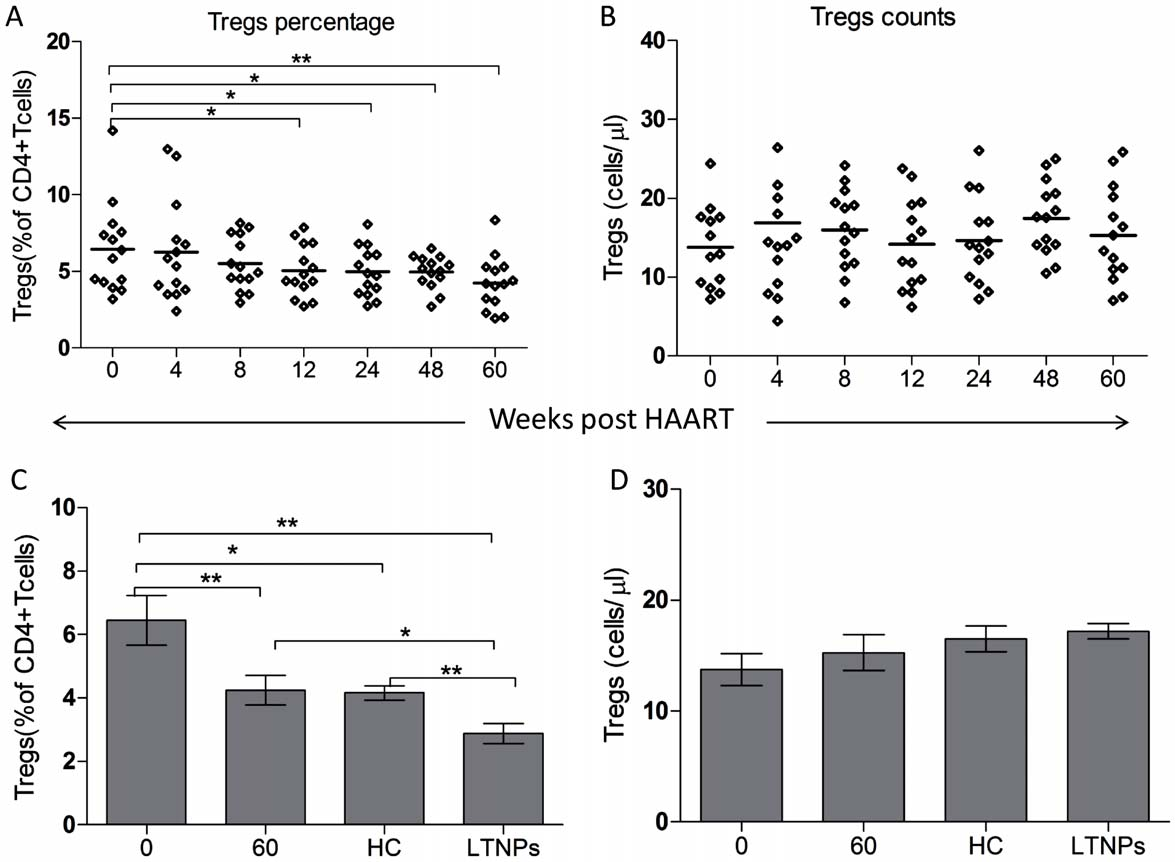
Longitudinal changes in the percentage and counts of Tregs during ART (weeks). (A–B) Longitudinal changes in the percentage and counts of Tregs in HIV-1-infected patients during ART. (C–D) Comparisons of the percentage and counts of Tregs among HIV-1-infected patients at week 0, week 60, healthy controls and LTNPs. **p<0.01, *p<0.05.

As shown in figure 3, the percentage was significantly reduced during ART, from 6.45% at w0 to 4.24% at w60 (6.45%, 6.26%, 5.51%, 5.04%, 4.97%, 4.95% and 4.24% at week 0, 4, 8, 12, 24, 48 and 60 respectively), and the corresponding *p*-values are 0.627, 0.102, 0.016, 0.048, 0.047, 0.001, compared to w0. In addition, the percentage of Tregs at w0 in HIV-1-infected patients was significantly higher than that in healthy controls (4.16%, *p* = 0.013) and LTNPs (2.88%, *p* = 0.001). After 60 weeks of ART, the Tregs percentage in HIV-1-infected patients was equal to that in the healthy controls (*p* = 0.862), but still lower than that in LTNPs (*p* = 0.021). Unexpectedly, the Tregs percentage in LTNPs is lower than the healthy controls (*p* = 0.002). And the Tregs counts in HIV-1-infected patients were not significantly changed during ART, ranging from 13.8 cells/ul to 17.4 cells/ul (13.8 cells/ul, 16.9 cells/ul, 15.9 cells/ul, 14.2 cells/ul, 17.4cells/ul and 15.3 cells/ul at week 0, 4, 8, 12, 24, 48 and 60 respectively; all *p*>0.05, compared to w0).

### Association between DCs counts and Tregs counts

**Figure 4:**
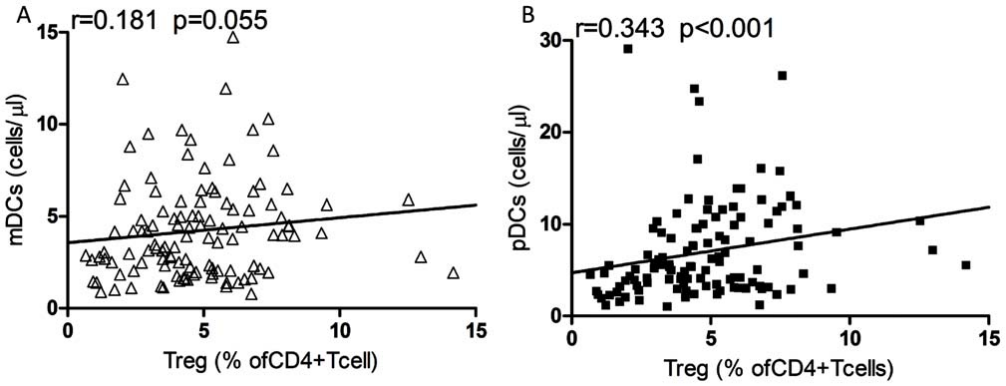
The association of DC subsets with Tregs percentage. (A) Correlation between mDCs counts and Tregs percentage during ART. (B) Correlation between pDCs counts and Tregs percentage during ART.

Interestingly, pDCs counts were positively associated with Tregs percentage (r = 0.343, *p*<0.001), but there were no correlations between mDCs counts and Tregs percentage (r = 0.181, *p* = 0.055) (Figure 4).

## Discussion

In the present study, we followed the counts of peripheral blood DC subsets, Tregs and IFN-a plasma levels in chronic HIV-1-infected patients over 60 weeks of ART,and compared with those in healthy controls and LTNPs. All chronic patients were given AZT+3TC+NVP or D4T+3TC+NVP, showing excellent adherence during treatment. As a result, a great curative effect of ART was achieved, with the viral loads falling below the limit of detection within 24 weeks, as well as CD4 counts increasing by approximately 150 cells/ul after 60 weeks of ART in all chronic HIV-1-infected patients.

We find that the impacts of ART on the counts and function of blood DC subsets in the HIV-1-infected patients are significantly different. The results indicated that mDCs counts in HIV-1-infected patients before ART were significantly lower than healthy controls, which were consistent with previous reports [9]–[13]. However, the mechanisms used by HIV to affect DCs are not fully understood. It could be due to HIV induced or HIV-related cell death. Blood mDCs counts began to increase early at 8 weeks post ART, reaching to the level of the healthy controls after 60 weeks. Our results confirm findings by other reports [11], [22]. In contrast, LTNPs had relatively normal levels of blood DCs [13]. After 60 weeks of ART, the mDCs counts in the chronic HIV-1-infected patients still lower than those in LTNPs, suggesting that mDCs may protect HIV-infected patients from progress to AIDS. The mechanisms that account for the increase in circulating mDCs after ART are likely due to the redistribution of mDCs from lymphoid tissue to blood. In addition, it is also possible that the total pool of mDCs rises slowly with continued suppression of viral replication, by way of either expansion of existing mDCs or thymic generation [23]. This conclusion is also supported by the findings that ART reduced activation of mDCs and expression of CCR7and caspase-3, and transiently restored mDCs counts in monkeys with progressive infection [9], [24], [25]. What' more, it is reported that the mDCs precursors increased above normal levels during chronic infection, particularly in LTNPs [13]. Conversely, Schmidt *et al.* [26] reported that no significant differences were observed in changes of absolute mDCs counts after 12-month ART.

In contrast to mDCs, our results showed that pDCs counts in HIV-1-infected patients did not decline before ART compared to those in healthy controls nor increased during ART. Even though there were no differences in pDCs counts between HIV-1-infected patients and healthy controls and LTNPs, in a more recent report, Geng *et al.* [27] showed that there were significantly lower pDCs counts in rapid progressors compared with typical progressors and healthy controls, suggesting that factors affecting pDCs counts were not just HIV. However, others described a reduction of pDCs counts in HIV-infected patients, and an impaired restoration of pDCs counts after ART [11], [22].

Reasons that account for the conflicting results of DCs counts may be as follows [10], [14], [22], [28]. (1) There was a great diversity among the study subjects. For example, some patients had high VLs and low CD4 counts before ART, while others were complicated with opportunistic infections or at different stages of disease progression. (2) Different DCs markers were used in different studies, such as HLA-DR+CD11c+Lin− [11], [15], CD4+CD11c+Lin− [26], [29], BDCA1+BDCA2− [22] for mDCs, and CD123^high^HLA-DR+Lin− [11], [15], CD4+CD11c−Lin− [26], [29], BDCA1−BDCA2+ [22] for pDCs. (3) There might be remarkable individual differences across studies, such as ethnics and treatment sensitivity. (4) The time to initiate ART, existence of HIV resistant strains, drug toxicity, and poor patient adherence to medication may affect DCs counts during ART. In addition, as relatively rare cells in peripheral blood, DCs also have higher variability. Thus DCs quantification requires the acquisition of large numbers of events and a rare event analysis. Many studies have indicated that DCs counts are of potential interest in clinical immunology, thus, standardization between the different laboratories involved is urgently needed.

IFN-a in vivo is mainly produced by pDCs, following interactions with HIV-infected cells or TLR ligands originated from gut microbial translocation [30]. Our observations showed that there was no decrease in IFN-a plasma levels in HIV-1-infected patients before ART or a change of IFN-a levels during ART, which was similar to the changes of pDCs counts and consistent with data reported by others [10], [29]. Nevertheless, there are also studies demonstrating that IFN-a production by virus stimulated PBMCs are reduced in HIV-infected patients [11], [15].The reason for discrepancy between them remains undetermined.

To understand whether the increase of mDCs is linked to plasma viral loads and CD4 counts during ART, we investigated the correlations among them. Our results showed that the chronic HIV-1-infected patients indeed had significantly positive correlation between mDCs counts and CD4 counts during ART. CD4 counts are usually considered as an indicator of preserved function of the immune system. The increase of mDCs counts after ART related to CD4 counts may also suggest the restoration of immune responses. It is notable that changes in mDCs at week 8 were negatively correlated with the changes in viral loads and positively correlated with the changes in CD4 counts at week 60 post ART, suggesting that mDCs counts at week 8 post ART are important for predicting the immune reconstitution and mDCs.

As just discussed for DCs, the impact of ART on the percentage and counts of Tregs in HIV-infected patients are also full of contradictions. We show that compared to the healthy controls, Tregs percentage in HIV-1-infected patients was significantly increased before ART, and decreased to normal levels after 60 weeks of ART. Additionally, LTNPs have the lowest frequency. These results are bolstered by recent studies showing that there was a significant higher percentage in patients with lower CD4 counts and ART reduced Tregs frequencies [17], [19], [31]. It is reported that Tregs are inversely correlated with activation of T cells [20], [32], [33], and elite controllers have lower Tregs frequencies compared to chronic HIV-infected patients, including patients on ART [33]. In this context, it is suggested that low Tregs may play a positive role in the HIV infection as they can maintain strong antiviral immune responses at the cost of hyperactivation. Mechanisms underlying loss of Tregs frequency during ART are not well understood, but could include Tregs redistribution and reduced thymic/virus-induced proliferation, or lower non-Treg cells death which indirectly caused the loss in Tregs percentage after ART [17], [33]–[35]. Nevertheless, some authors observed that there was an increase or no change in Tregs percentage during ART [18], [21]. Additionally, Jiao *et al*. [20] demonstrated that efficient ART resulted in Treg frequency normalizing in complete responders to the treatment but not in non-responders. In contrast, Tregs counts in HIV-1-infected patients before ART were not different from healthy controls [16], and were not changed during ART. Interestingly, other studies suggested that there was an increase or a decrease in Tregs counts after ART [17], [18], [21]. Numerous factors such as the timing and intensity of ART and methodological discrepancies of the Tregs detection have considerable impacts on the changes of Tregs during ART. The findings in this context are very preliminary and much more detailed investigations need to be carried out.

There is accumulating evidence that DCs and Tregs are interacting with each other by cell-cell contacts and soluble factors [36], [37]. For example, it is suggested that interactions between HIV and DCs lead to the development of semimature DCs which induce Tregs production, while these semimature DCs disappearance correlates with the initiation of ART [35]. Another study reported that HIV-stimulated pDCs could induce Tregs production by expression of indoleamine 2, 3-dioxygenase, and the Tregs induced by pDCs were shown to inhibit the maturation of bystander conventional DCs [38]. However, the role of the interplay between DCs and Tregs in HIV infection still needs to be clarified. Interestingly, we showed that pDCs counts were positively associated with Tregs percentage during ART.

The present study examined the longitudinal changes of peripheral blood DC subsets and Tregs in a group of chronically asymptomatic treatment-naive HIV-1-infected patients over 60 weeks of ART, and compared with those in healthy controls and LTNPs. Although the mechanisms responsible for the observed impact of ART on them remain undefined, our data indicate that mDCs counts reduced in chronic HIV-1-infected patients and normalize after 60 weeks of ART but still lower than those in LTNPs, together with fully suppressed VLs and increased CD4 counts. However, the counts and function of pDCs were relatively stable during ART. Our longitudinal data from ART-treated subjects suggest that the mDCs defects may be reversible and mDCs (not pDCs) are likely important APCs that bridge the gap between innate and adaptive immunity in HIV infection. What's more, Tregs percentage increased before ART and normalized after ART, suggesting that low Tregs percentage may benefit antiviral immune responses in HIV infection. Information regarding the dynamics of DC subsets and Tregs during ART may improve our understanding of the role of these cells in HIV infection.

## Materials and Methods

### Subjects

There were 17 chronically asymptomatic treatment-naive HIV-1-infected patients enrolled in our study. Inclusion criteria for the HIV-1+group included CD4 counts less than 350cells/ul and no-opportunistic infection within the previous three months. Patients with pregnancy, active tuberculosis, acute infectious diseases or serious liver/renal dysfunction were excluded from the study. All individuals received ART, which included two nucleoside reverse-transcriptase inhibitors (NRTIs) plus one non-nucleoside reverse transcriptase inhibitor (NNRTI) (Table 1). After 24 weeks of ART, VLs in almost all patients were below the lower detection limit. Additional 15 HIV-uninfected age-matched healthy adults and 15 long term non-progressors (LTNPs) were recruited as controls. The definition of LTNPs was HIV-infected for greater than 10 years with lower detectable viremia and high CD4 counts without ART. Blood was collected at week 0, 4, 12, 24, 48 and 60 of treatment. All subjects were from Beijing You'an Hospital, Capital Medical University. The study was approved by the Beijing You'an Hospital Research Ethics Committee, and written informed consent was obtained from each subject.

### Enumeration of DC Subsets and Tregs in PBMCs

PBMCs were isolated by density gradient centrifugation using Ficoll-Hypaque (Amersham Pharmacia Biotech, Piscataway, NJ) from whole blood. Four-color flow cytometric analysis was performed using FACS Calibur (BD Biosciences, San Diego, USA) and data of DCs and Tregs were collected using identical instrument settings. All data obtained was analyzed using FlowJo (Treestar, Ashland, OR). The counts of blood DCs and Tregs were calculated using the dual platform TruCOUNT assay (see below).

**Figure 5:**
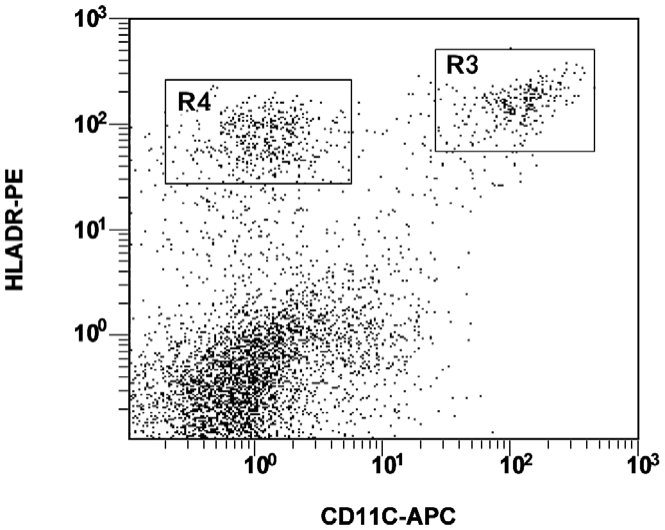
Rare event analysis of DC subsets by flow cytometry: mDCs (R3) and pDCs (R4).

To identify DCs, the following antibodies from BD Pharmingen (San Diego, USA) were used: CD4-PerCP, Lin-FITC, HLA-Dr-PE and CD11c-APC. At least 200,000 events were acquired for each sample. mDCs were identified as Lin−HLA-DR+CD11c+, while pDCs were Lin-HLA−DR+CD11c− [26], [39], [40]. DCs counts were calculated as follows, utilizing hemocytometer data for lymphocytes and monocytes and flow cytometry data for windows [10]: mDCs/pDCs per ul of blood = counts of (monocytes+lymphocytes)/ul× (number of events in the population [ie, in R3 or R4, Figure 5]/number of PBMCs).

**Figure 6:**
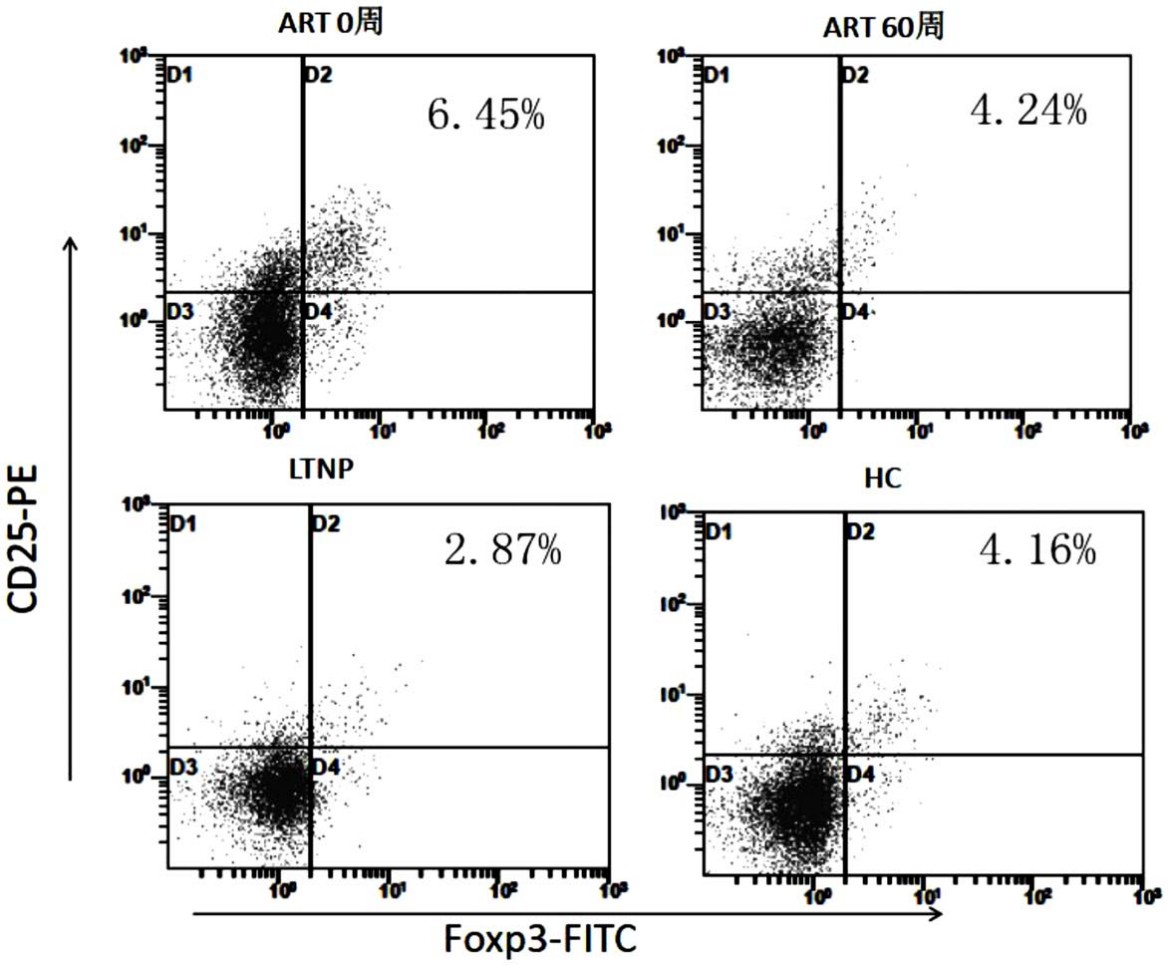
Representative plots of CD4+CD25+FoxP3+Tregs from individual subjects in the HIV-1-infected patients at week 0, week 60, healthy controls and LTNPs.

For staining of Tregs, the cells were first stained with CD3-PerCP, CD4-FITC and CD25-PE (BD Pharmingen, San Diego, USA), then permeabilized and fixed using eBioscience fix/perm (eBioscience, San Diego, USA) according to the manufacturer's instructions. After 30-minute permeabilization, FoxP3-FITC (eBioscience, San Diego, USA) was added for another 30 minutes. Tregs were identified as CD3+CD4+CD25+Foxp3+. The counts of Tregs were deduced from the CD4+T cells counts multiplied by the proportion of Tregs in CD4+T cells (Figure 6).

### IFN-a plasma level Analysis

The IFN-a plasma levels were detected using a human IFN-a ELISA kit and performed according to the manufacture's instruction (Mabtech, USA). Sensitivity of the assay was 10-3160 pg/ml. The limit of detection of this assay is 3 pg/ml.

### Viral Load

Plasma HIV-1 RNA was quantified by real time-PCR (Roche, Germany) and the sensitivity of detection of this super sensitive assay was 40 copies/ml. Values below the limits of detection were treated as 20 copies/ml.

### Statistical analysis

Data analysis was performed with SPSS 16 software (SPSS Inc, Chicago, IL). The Mann-Whitney test was utilized for comparisons between two independent groups. The Wilcoxon signed rank test was used for comparisons within subjects. The correlations between variables were calculated using the Spearman's rank correlation test and linear regression analysis were plotted using the Graph Pad Prism software (v 5.0). All statistical analyses assumed a two-sided significance at *p* values<0.05.
